# Genome-Wide Analysis of MYB Transcription Factors in the Wheat Genome and Their Roles in Salt Stress Response

**DOI:** 10.3390/cells12101431

**Published:** 2023-05-20

**Authors:** Selvakumar Sukumaran, Johanna Lethin, Xin Liu, Justyna Pelc, Peng Zeng, Sameer Hassan, Henrik Aronsson

**Affiliations:** 1 Department of Biological and Environment Sciences, University of Gothenburg, 405 30 Gothenburg, Sweden; 2 Triticeae Research Institute, Sichuan Agricultural University, Wenjiang 611130, China; 3 Department of Bioengineering, Faculty of Environmental Management and Agriculture, West Pomeranian University of Technology, 71-434 Szczecin, Poland; 4 State Key Laboratory of Crop Genetics and Germplasm Enhancement, Nanjing Agricultural University, Nanjing 210014, China; 5 OlsAro Crop Biotech AB, Erik Dahlbergsgatan 11A, 41126 Gothenburg, Sweden

**Keywords:** BARI Gom-25, MYB, salinity, salt stress, transcription factor, wheat

## Abstract

Large and rapidly increasing areas of salt-affected soils are posing major challenges for the agricultural sector. Most fields used for the important food crop *Triticum aestivum* (wheat) are expected to be salt-affected within 50 years. To counter the associated problems, it is essential to understand the molecular mechanisms involved in salt stress responses and tolerance, thereby enabling their exploitation in the development of salt-tolerant varieties. The myeloblastosis (MYB) family of transcription factors are key regulators of responses to both biotic and abiotic stress, including salt stress. Thus, we used the Chinese spring wheat genome assembled by the International Wheat Genome Sequencing Consortium to identify putative MYB proteins (719 in total). Protein families (PFAM) analysis of the MYB sequences identified 28 combinations of 16 domains in the encoded proteins. The most common consisted of MYB_DNA-binding and MYB-DNA-bind_6 domains, and five highly conserved tryptophans were located in the aligned MYB protein sequence. Interestingly, we found and characterized a novel 5R-MYB group in the wheat genome. In silico studies showed that MYB transcription factors MYB3, MYB4, MYB13 and MYB59 are involved in salt stress responses. qPCR analysis confirmed upregulation of the expression of all these MYBs in both roots and shoots of the wheat variety BARI Gom-25 (except MYB4, which was downregulated in roots) under salt stress. Moreover, we identified nine target genes involved in salt stress that are regulated by the four MYB proteins, most of which have cellular locations and are involved in catalytic and binding activities associated with various cellular and metabolic processes.

## 1. Introduction

Climate change has put pressure on agriculture to continue to preserve a high food production level to feed the world’s population despite challenges such as increased negative stresses to plants. Thus, conditions favourable for domesticated crops have started to decline as these crops are prone to stresses, e.g., cold, heat, drought and salt [1]. Salt stress is particularly challenging because salt-affected soils (2 dS/m) are present in more than 100 countries [2] and have been recently shown to cover 833 Mha of the global area [3]. With the current rate of global warming, it is predicted that almost 50% of fields might be unproductive 50 years from now [4,5]. One of the susceptible crops, wheat, is an essential food and commercial crop globally, and similar to various other glycophytes, it lacks mechanisms that enable survival in highly saline conditions [6,7]. Salt stress affects plants in two phases: an osmotic phase involving osmotic imbalance and impairment of shoot growth and an ionic phase involving accumulation of ions to toxic levels [8]. Osmotic effects occur rapidly after stress induction, while ionic stress develops more slowly after persistent stress [9,10,11]. High soil salinity inhibits plant growth and development by reducing water uptake, which results in the accumulation of toxic ions and reductions in nutrient availability, further leading to ion imbalance and toxicity that inhibit enzymatic reactions in vital biological processes [12,13]. 

Salt stress tolerance mechanisms in plants involve recognition of environmental signals by receptors located in cell wall membranes, signal transduction and transcription factor-mediated regulation of genes involved in responses that enable plants to survive in the presence of salinity up to taxa-specific thresholds [14]. Previous studies have shown that wheat has a highly complex genome, including three subgenomes (A, B and D), which has still not been fully characterized, complicating efforts to pinpoint all the genes involved in its salt tolerance [15,16] or other stress responses. However, the myeloblastis (MYB) transcription factor family is known to have significant effects on salt tolerance in wheat [17]. The MYB proteins consist of a vast group of transcription factors with a diverse set of functions. For example, they are key regulatory elements in development, metabolism, responses to biotic and abiotic stresses and disease resistance. So far, 126, 118, 1326 and 155 MYB genes have been reported in *Arabidopsis thaliana* (Arabidopsis) [18], *Camellia sinensis* [18], *Solanaceae* species [19] and *Orzya sativa* (rice) [20], respectively. Although MYB proteins are involved in diverse processes in plants, all their functions depend on the presence of an MYB domain repeat, which confers the ability to bind DNA and thereby regulate the expression of other functional genes [21]. 

MYB protein domains have 51–53 amino acids (https://phytozome-next.jgi.doe.gov/phytomine/report.do?id=3298492, accessed on 19 January 2020), adjacent imperfect repeats in their DNA-binding domains and are classified into four [20] subfamilies: R1/2-MYB, R2R3-MYB, R1R2R3-MYB (3R-MYB) and 4R-MYB (containing four R1/R2-like repeats). Early studies showed that R2R3-MYB is the largest family, and its members are involved in salt stress responses [22,23]. Three α-helices form the MYB domain. The second and third create a helix-turn-helix structure that introduces a groove, which enables MYB proteins to bind with DNA [21]. Abundant R2R3-MYB proteins have been categorised by genetic approaches and found to be involved in the control of plant-specific processes, including plants’ development, growth, metabolism, stress responses, secondary metabolism, cell morphogenesis, leaf senescence, chloroplast development, responses to phosphate starvation and tolerance of drought, cold and salt stress [21,24,25]. Therefore, in attempts to facilitate elucidation and improvement of wheat’s salt tolerance, we have examined MYB genes (full coding sequence of that loci) and domains (part of the full coding sequence) and the genes’ expression in this crucial crop plant, as described in the following sections.

## 2. Materials and Methods

### 2.1. Identification of MYB Sequences

The Hidden Markov Model file of the wheat domain (PF00249) was downloaded from the PFAM database to identify sequences in the MYB genome (PFAM version 32.0). To match the MYB-sequences with the wheat genome, the proteome of the *Triticum aestivum* cultivar Chinese spring genome was downloaded from the Ensemble plants database (ftp://ftp.ensemblgenomes.org/pub/plants/release41/fasta/triticum_aestivum/pep/, accessed on 10 March 2020) and used as a reference. The HMM profile of the MYB domain was used as a query to scan the wheat proteome using HMMER software (Version 3.1) with an E value of 1 × 10^−5^. An in-house Python script was used to extract amino acid sequences of the MYB domain-containing proteins in the wheat proteome. The redundant protein sequences were identified using CD-HIT (http://weizhong-lab.ucsd.edu/cdhit-web-server/cgi-bin/index.cgi, accessed on 3 May 2020) with a sequence identity cut-off of 100%, and representative sequences were used for further analysis.

### 2.2. Transcription Factor Binding Site Prediction

The upstream regions (2000 bp) of all the genes were extracted from the IWGSC Chinese spring wheat genome accessed via the Ensembl database. The Bedtools getfasta option was used to extract upstream sequences of the individual genes. The MYB protein sequences were subjected to profile inference tool analysis to identify their JASPAR transcription factor-binding profiles. An in-house Python script (https://github.com/Sameerpython/Transcription-Factors, accessed on 10 June 2020) was written to search the JASPAR database. This identified 13 reported MYB transcription factor profiles. Following an analysis using Genevestigator (www.genevestigator.com, accessed on 28 June 2020) of their roles and expression related to salinity stress, a Position Weight Matrix (PWM) of four selected MYB sequences (MYB3, MYB4, MYB13 and MYB59) was downloaded in MEME format from the JASPAR CORE database. The upstream region of every gene was then scanned against the four PWMs to predict its MYB transcription binding site with a *p*-value < 1 × 10^−5^ using the FIMO tool (http://meme-suite.org/index.html, accessed on 16 May 2021). The top 3000 gene IDs from FIMO targeted by each of the MYB3, MYB4, MYB13 and MYB59 transcription factor families were identified and screened using R for the 144 genes previously identified as involved in salt stress [26]. Furthermore, the gene onthology (GO) was assigned for the MYB target genes using WEGO (www.wego.genomics.cn, accessed on 20 June 2021) [27]. 

### 2.3. Phylogenetic Analysis

The protein sequences of MYB domain from 719 Myb genes were aligned using the ClustalO multiple sequence alignment server. The aligned sequences were then converted into phylip format for building a phylogenetic tree using the RAxML software [28]. RAxML HPC (v8.2.10) was employed for constructing the phylogenetic tree using the PROTGAMMAAUTO model with 100 times bootstrap iterations. The tree shown in this study was visualized using the Interactive Tree of Life (iTOL) server. The sequences were assigned to different groups based on previous studies [21,29]. 

### 2.4. Plant Growth Conditions and Treatments

BARI Gom-25 wheat seeds were germinated on wet Munktell A1-100-80^TM^ filter paper for three days in darkness at room temperature. Resulting seedlings were transferred to a hydroponic growth system containing continuously aerated tap water mixed with Nelson Garden Hydroponic Nutrition^TM^ (Nelson Garden, Tingsryd, Sweden) (2 mL/l) at the start of the fourth day. After 6 days of hydroponic growth, 100 mM NaCl salt was added to 1 unit of the system to induce salt stress, while the medium was not changed in another unit to provide unstressed controls. After 6 days, shoot and root parts of plants subjected to salt stress and controls were separately harvested, instantly frozen in liquid nitrogen and stored at −80 °C until further analyses. Each root or shoot sample included pooled tissues of 20 plants.

### 2.5. Quantitative Real-Time PCR (qPCR)

Frozen plant shoot and root materials were pulverised using a MM 301 Mixer Mill (Retsch GmBH) twice for 15 sec. Total RNA was extracted from the tissues following the manual of the NucleoSpin RNA Plant^TM^ kit (Macherey-Nagel, Düren, Germany). An iScript^TM^ cDNA Synthesis Kit (Bio-Rad, Hercules, USA) was used to synthesize cDNA from the root and shoot samples using 100 ng portions of total RNA, which were subjected to qPCR analyses using a BioRad CFX96 Real Time^TM^ system following instructions in a SsoAdvanced Universal SYBR Green Supermix^TM^ (Bio-Rad, Hercules, USA) for qPCR manual. Changes in expression of genes of interest were analysed using the 2^–ΔΔCt^ method and the BARI Gom-25 actin gene as a reference housekeeping gene with three replicates of each qPCR sample and three repetitions of all experiments. Primers used are listed in Supplementary Table S1. Primers were designed using the Primer 5 software (Premier Biosoft, Palo Alto, CA, USA) with the following selection criteria: span two exons, GC content of 55–65%, 70–120 bp of qPCR products and Tm between 59–62 °C.

## 3. Results

### 3.1. Identification of MYB Transcription Factors in Wheat

A genome-wide identification of the MYB transcription factor gene family was performed using the HMMER software against the recently updated wheat genome sequence. The analysis resulted in the identification of 719 MYB genes and 924 MYB transcript isoform sequences. Our analysis resulted in identifying 243, 234 and 241 MYB genes in A, B and D subgenomes, respectively. One MYB gene was identified in the Unknown chromosome as defined in the Chinese wheat spring genome. Thus, the subgenome A was observed to have the highest number of MYB genes in the wheat genome. A total of 290 1R-MYB, 412 2R-MYB, 14 3R-MYB, 1 4R-MYB and 2 5R-MYB were identified in the wheat genome.

**Figure 1:**
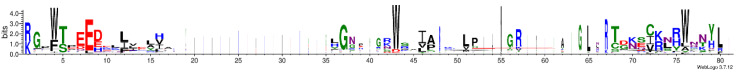
WebLogo analysis of the MYB domain sequences. Graphical representation of the multiple sequence alignments of MYB transcription factor domains identified in wheat. Black, hydrophobic amino acid; green, polar amino acid; blue, positively charged amino acid; red, negatively charged amino acid; purple, neutral amino acid. Bits, conservation at that amino acid position.

PFAM analysis of the MYB sequences identified 28 combinations (Supplementary Table S2) of 16 domains (Supplementary Table S3), including 6 MYB-related domains. The most frequent of the 28 combinations (detected in 363 genes), designated Group 1, includes MYB_DNA-binding, MYB_DNA-binding, MYB_DNA-bind_6 and MYB_DNA-bind_6 domains. In 8 of the 28 groups, the MYB_DNA-binding domain is present at least twice. In Groups 23 and 26, there are five repetitions of the MYB_DNA-binding domain. The most frequently observed domain after MYB_DNA-binding in the 28 groups is MYB_DNA-bind_6 (in 19 groups). The most frequently observed non-MYB domains along with MYB_DNA-binding are the SWIRM domains (in four groups). The SWIRM domain has a helix-turn-helix motif and binds to DNA [30]. The WebLogo for the identified MYB sequences shows that tryptophan residues at three positions are very highly conserved (Figure 1). In addition to detecting proteins with multi-domain MYB architecture, we also identified 134 members of the 1R-MYB group with just a single MYB domain. 

### 3.2. Phylogenetic Tree

**Figure 2:**
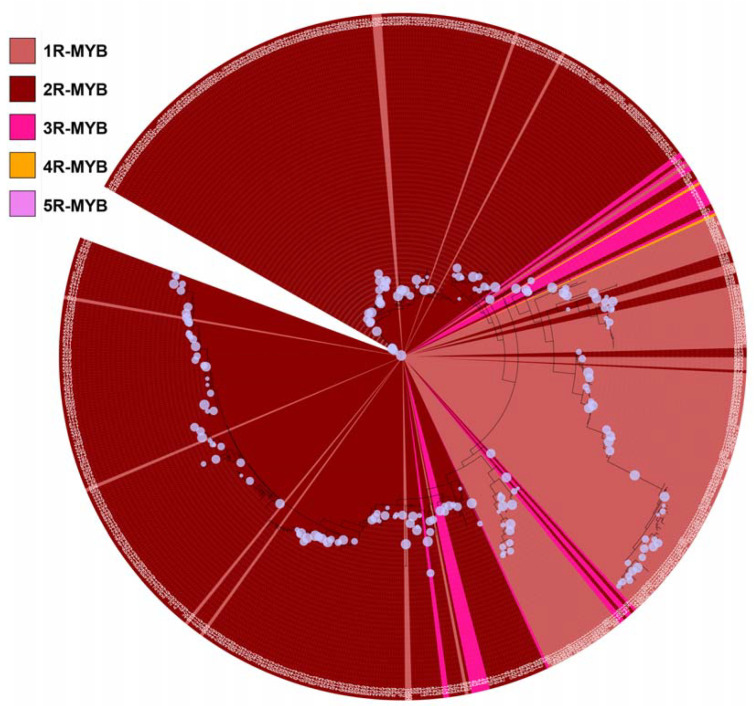
Phylogenetic tree of MYB transcription factor subfamily proteins in wheat. The tree was constructed for the 5 MYB R groups using a total amount of 719 sequences of wheat cv Chinese spring (Supplementary Tables S4 and S5). The light brown, brown, pink, orange and purple zones represent 290 1R-MYB, 412 2R-MYB; 14 3R-MYB; 1 4R-MYB and 2 5R-MYB protein sequences. The numbers surrounding the tree are the corresponding 1170 MYB domain sequences giving rise to the 5 MYB R groups. The phylogenetic tree file can be assessed from Supplementary Table S6.

The evolutionary relationships of 1170 identified MYB domain sequences from 719 genes were analysed using RAxML. We found distinct clustering of the 1R-MYB group and the 2R-MYB (R2-R3) group except for a few MYB domains (Figure 2). However, the analysis also revealed nested clades of the 3R-MYB, 4R-MYB and 5R-MYB groups within the 1R-MYB group and a few dispersed 1R-MYB genes within the clades of the 2R-MYB genes, 3R-MYB genes with 2R-MYB genes and 2R-MYB genes with the 1R-MYB genes (Figure 2). 

### 3.3. Structure-Based Analysis of MYB Transcription Factors

**Figure 3:**
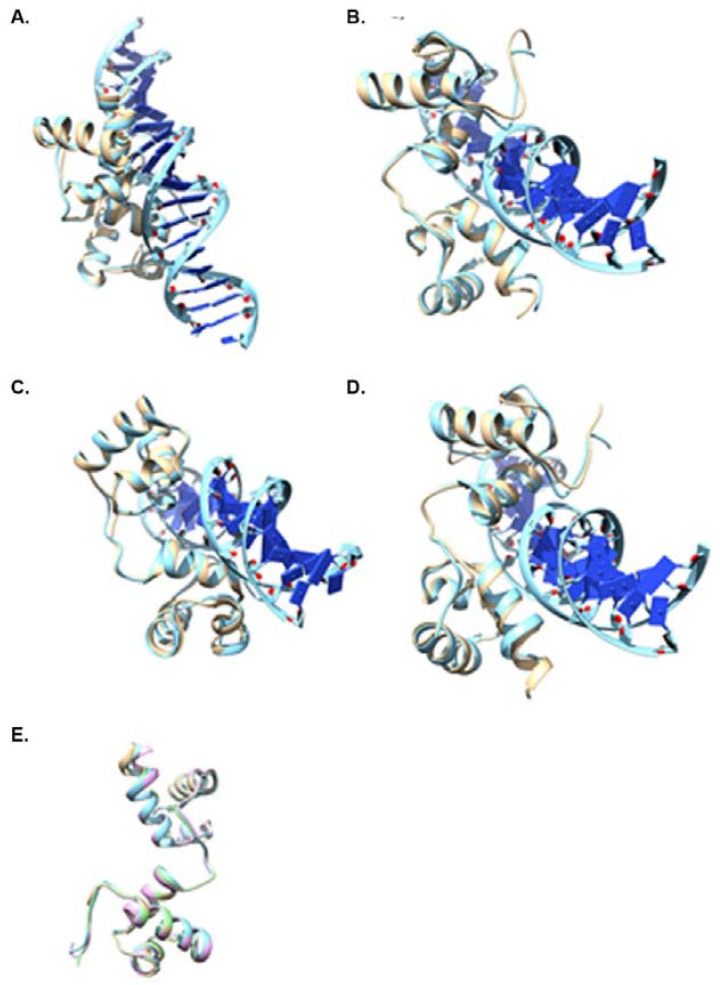
Predicted model structures of MYB3, MYB4, MYB13 and MYB59. Structures predicted using AlphaFold and superimposed with the MYB (PDB ID: 6KKS) crystal structure coloured blue. A. MYB3, B. MYB4, C. MYB13, D. MYB59 and E. structural superimposition of MYB3 (blue), MYB4 (pink), MYB13 (brown) and MYB59 (green).

We predicted three-dimensional structures of four MYB proteins that are apparently involved in salt tolerance (MYB3, MYB4, MYB13 and MYB59) using AlphaFold and sequences downloaded from the Ensembl database. All four of these proteins were identified as belonging to the 2R-MYB group based on the phylogenetic tree (Figure 2), supporting previous data [25]. Relaxed models for the four proteins generated by AlphaFold were selected, submitted for fold search analysis (Table 1) and found to have fold similarity to an Arabidopsis R2R3-type MYB transcription factor (Uniprot ID, Q9SEI0; PDB ID, 6KKS). Interestingly, fold comparison analysis revealed structural similarity of the MYB domains to the human DNA-binding cell division cycle 5-like protein involved in cell division, which may act as a transcription activator (Uniprot ID, Q99459; PDB ID, 7DVQ chain L). Thus, the four MYB proteins are predicted to have a very conserved fold (Figure 3).

**Figure 4:**
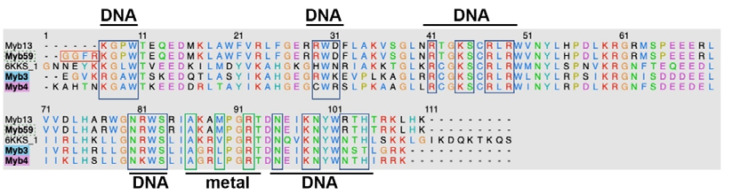
Multiple sequence alignment of the wheat MYB sequences with the sequence of the crystal structure of *Arabidopsis thaliana* R2R3-type MYB transcription factor (PDB ID: 6KKS). Blue and green boxes indicate the DNA-binding amino acids and metal-binding sites, respectively.

To map the DNA-binding amino acids of domains of the four MYB transcription factors (MYB3, MYB4, MYB13 and MYB59), we aligned their protein domain sequences against the Arabidopsis R2R3-type MYB transcription factor (PDB ID: 6KKS) bound to a DNA recognition sequence (Figure 4). The DNA binding amino acids in 6KKS were identified from the PDBsum database. The alignment indicates that the binding site is well conserved in the MYB domains of MYB3, MYB4, MYB13 and MYB59. Amino acids at 16 of the 23 positions mapped as the DNA-binding site are identical, corroborating the high conservation at these positions. Of the three amino acids, two (alanine and arginine) binding to the metal are identical in all four MYB sequences. 

### 3.4. MYB Transcription Factor Expression under Salt Stress 

**Figure 5:**
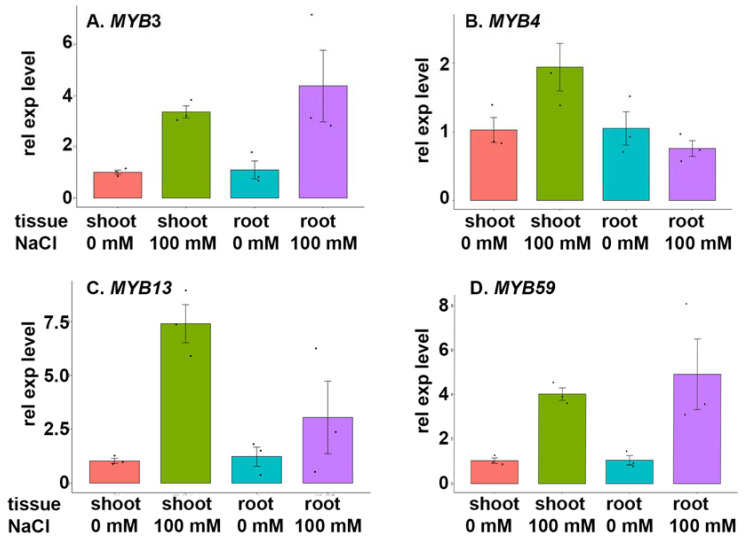
Expression of *MYB* transcription factors under salt stress (100 mM NaCl) and in unstressed controls. A hydroponic system was used to grow BARI Gom-25 seedlings for 6 days, and then 100 mM NaCl was added to the medium (except for controls). Six days later, shoots and roots were harvested and analysed to assess changes in relative expression level (rel exp level) of (**A**). *MYB3*, (**B**). *MYB4*, (**C**). *MYB13* and (**D**). *MYB59*.

To study the expression of the 4 identified MYB transcription factors, BARI Gom-25 seedlings were grown in a hydroponic system for 6 days without salt, and then 100 mM NaCl was added to the medium, and they were sampled 6 days later. As shown in Figure 5, expression levels of *MYB3*, *MYB4*, *MYB13* and *MYB59* genes were higher in shoots following the 100 mM NaCl addition than in the preceding, unstressed conditions and controls. Expression levels of *MYB3*, *MYB13* and *MYB59* were also higher under salt stress in roots than in controls, but *MYB4* expression was lower. The greatest observed increase in shoots under salt stress was of *MYB13* expression (seven-fold), but all the other increases in shoots were at least two-fold (Figure 5). In roots, *MYB59* expression was most strongly increased (ca. 5-fold), followed by *MYB3* and *MYB13* (4-fold and 2-fold, respectively), while *MYB4* expression was ca. 25% lower (Figure 5).

### 3.5. Target Genes Involved in Salt Stress That Are Regulated by MYB Transcription Factors

A mapping of 144 genes known to be expressed during salt stress to genes identified by FIMO of each of the 4 MYB families (MYB3, MYB4, MYB13 and MYB59) identified nine induced by salt stress as target genes for these MYB transcription factors (Table 2). Although all can be related to a family of genes, five of these nine genes encode uncharacterized proteins (Table 2). MYB3 regulates two of the genes, but they are both also regulated by other MYBs (MYB4 and MYB59, respectively; Table 2). In addition, MYB13, MYB4 and MYB59, respectively, regulate two, three and four of the genes (Table 2). 

**Figure 6:**
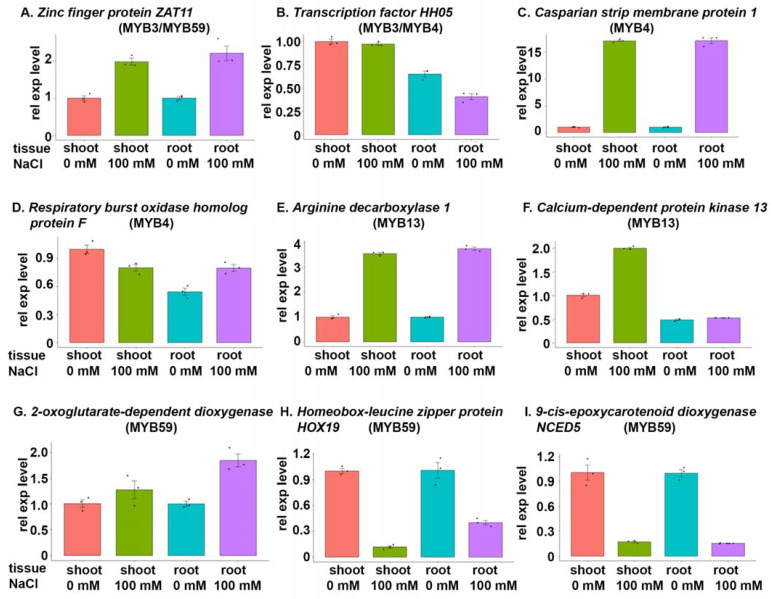
Expression of target genes regulated by MYB transcription factors under salt stress (100 mM NaCl) and in unstressed controls. A hydroponic system was used to grow BARI Gom-25 seedlings for 6 days, and then 100 mM NaCl was added to the medium (except for controls). Six days later, shoots and roots were harvested and analysed to assess changes in relative expression level (rel exp level) of target genes regulated by MYB transcription factors: MYB3 (**A**,**B**); MYB4 (**B**–**D**); MYB13 (**E**,**F**) and MYB59 (**A**,**G**–**I**). Target genes: (**A**) *zinc finger protein ZAT11*; (**B**) *transcription factor HHO5*; (**C**) *Casparian strip membrane protein 1*; (**D**) *respiratory burst oxidase homolog protein F*; (**E**) *arginine decarboxylase 1*; (**F**) *calcium-dependent protein kinase 13*; (**G***) 2-oxoglutarate-dependent dioxygenase*; (**H**) *homeobox-leucine zipper protein HOX19*; (**I**) *9-cis-epoxycarotenoid dioxygenase NCED5*.

Expression patterns of the two genes regulated by MYB3 changed following exposure to 100 mM NaCl; expression of the *zinc finger protein ZAT11* increased approximately 2-fold in both shoots and roots, while expression of *transcription factor HHO5* did not clearly change in shoots but declined by approximately 40% in roots (Figure 6A,B).

The expression pattern of *transcription factor HHO5* is also valid for MYB4 as a regulator (Figure 6B). Effects of salt stress on expression patterns of the other two genes regulated by MYB4 included strong (approximately fifteen-fold) upregulation of the *Casparian strip membrane protein 1* and a decrease in expression of the *respiratory burst oxidase homolog protein F* in shoots accompanied by an increase in its expression in roots (Figure 6C,D). 

Expression of the *arginine decarboyxlase 1* target gene of MYB13 increased more than three-fold under salt stress (100 mM NaCl), whereas expression of the second target gene of MYB13 encoding *calcium-dependent protein kinase* increased in shoots under salt stress but decreased in roots (Figure 6E,F). 

MYB59 regulated four target genes, and the expression of two of them, encoding *zinc finger protein ZAT11* and *2-oxoglutarate-dependent dioxygenase*, increased in both shoot and root tissues under salt stress (Figure 6A,G). In contrast, expression of the other two, encoding the *homeobox-leucine zipper protein HOX9* and *9-cis-epoxycarotenoid dioxygenase NCED5* decreased at least three-fold in both root and shoot tissues (Figure 6H,I).

**Figure 7:**
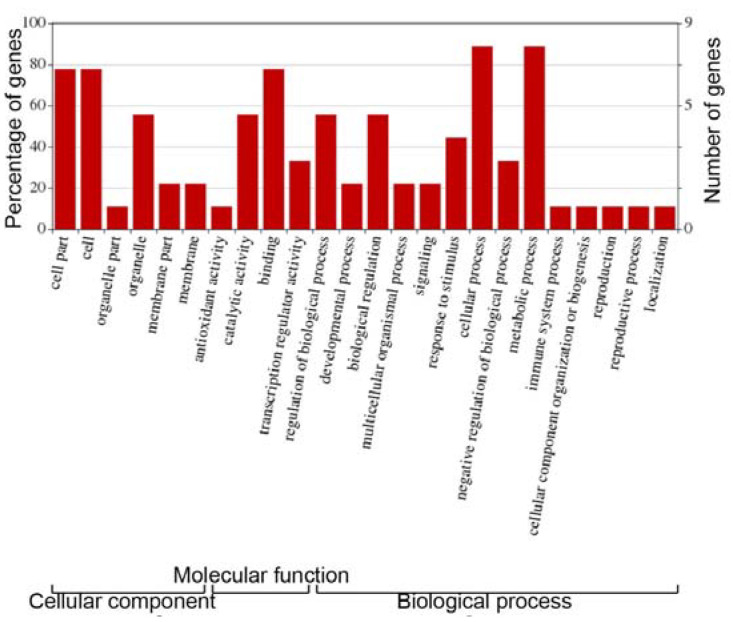
Results of gene ontology analysis of the nine genes involved in salt stress predicted to be regulated by MYB proteins. The results are classified into three categories: cellular component, molecular function and biological process. The left and right y-axes, respectively, show the percentage and number of the nine genes involved in each of the components, functions or processes.

Gene Ontology analysis of the MYB genes identified associations with several GO terms (Figure 7) and “Biological processes”, including positive regulation of the response to salt stress, regulation of stomatal movement, response to water deprivation, negative regulation of gene expression and regulation of root development. This clearly suggests that genes play important roles in adaptation to abiotic stresses, such as salt stress. The GO ID linked to positive regulation of responses to salt stress, and water deprivation mapped to the target gene *TraesCS5D02G411800*, *transcription factor HHO5*. *Transcription factor HHO5* has a recognized association with the negative regulation of gene expression, while the target gene *TraesCS3D02G350100*, *the zinc finger protein ZAT11*, is associated with regulation of root development. 

## 4. Discussion

The number (719) of MYB transcription factor genes we identified in wheat is around six- and five-fold higher than previously reported numbers in Arabidopsis and rice, respectively [18,20]. However, the hexaploidy genome of wheat (ca. 16 000 Mb) is much larger than the diploid genomes of Arabidopsis and rice (ca. 125 and 466 Mb, respectively). Thus, the differences in identified MYB gene numbers do not fully reflect the differences in genome size (128- and 34-fold, respectively). Whether this is due to differences in other mechanisms or domain architectures among the plants is unclear, but there is clear functional diversity in MYBs among these and many other plants [18,19,20].

The MYB protein sequences were divided into five MYB R groups, and the major R-group is the 2R-MYB group (412 sequences) followed by the 1R-MYB group (290 sequences) (Figure 2). This is consistent with previous findings [21,29]. In total, 393 R2-MYB and 12 R3-MYB genes have been reported in the wheat genome [25]. Our four identified MYB proteins all belong to the 2R-MYB group. The 5R-MYB group (2 sequences) is not very common, i.e., so far, it is only identified in a few species [29,32,33]. No members of this group have been previously reported in wheat, and further in-depth studies are needed to elucidate its role in wheat. However, in other organisms, up to a 6R-MYB protein have been reported [29]. 

When looking at the identified MYB sequences, one could observe very highly conserved tryptophan residues at three positions (Figure 1). These tryptophans are present in the R2 and R3 domains where they have known importance for the sequence specificity of DNA binding [34,35]. As observed in members of the *Solanaceae* family [19], five tryptophans are present in these domains in the wheat genome we examined: three in the R2 domain and two in the R3 domain. However, another potential tryptophan in R3 is replaced with a hydrophobic amino acid in *Solanaceae*. In addition, more than 30 years ago, it was proposed that a three-tryptophan cluster in MYB domains is strongly linked to DNA binding specificity [35]. 

The MYB multi-domain architectures consisted of 28 groups with 1 dominant group including more than 50% of the MYB protein sequences; 363 of 719 were identified in total (Supplementary Table S2). This was consistent with expectations, as the group contains the architecture of the common 2R-MYB group (with R2R3 domains). Most (17 out of 28) of the identified groups had only three or fewer members (Supplementary Table S2). Several MYB proteins with the common R2R3 architecture have been assigned functions, based on genetic findings, and linked to the regulation of primary and secondary metabolism, cell differentiation, developmental processes and both abiotic and biotic stress responses (Dubos et al., 2010). However, we cannot exclude the possibility that the groups with few members could have specialized functions in the wheat variety selected for our study. Clearly, there is high diversity in the structure and functions of MYB and hence the genes that they regulate (Dubos et al., 2010, Zeng et al., 2021). In our study, we focused on those that participate in responses to the abiotic stress of salinity. The MYB multi-domain architectures consisted of 28 groups with 1 dominant group including more than 50% of the MYB protein sequences; 363 of 719 were identified in total (Supplementary Table S2). This was consistent with expectations, as the group contains the architecture of the common 2R-MYB group (with R2R3 domains). Most (17 out of 28) of the identified groups had only three or fewer members (Supplementary Table S2). Several MYB proteins with the common R2R3 architecture have been assigned functions, based on genetic findings, and linked to the regulation of primary and secondary metabolism, cell differentiation, developmental processes and both abiotic and biotic stress responses [21]. However, we cannot exclude the possibility that the groups with few members could have specialized functions in the wheat variety selected for our study. Clearly, there is high diversity in the structure and functions of MYB and hence the genes that they regulate [21,29]. In our study, we focused on those that participate in responses to the abiotic stress of salinity. 

Except for our observation of increased expression of *MYB3*, *MYB4*, *MYB13* and *MYB59* during salt stress, increases in the expression of other MYB genes have been observed. Increased expression of MYB in seedlings under salt stress has been observed, e.g., *FtMYB9* in *Fagopyrum tataricum* (tartary buckwheat) [36]. Similarly, overexpression of some MYBs can strengthen salt tolerance, e.g., *MYB49* in tomato [37], *MYB32* in rice [38] and *GmMYB12B2* in Arabidopsis [39]. However, some MYBs can also be downregulated by salt, e.g., *VcMYB4a* in *Vaccinium caesariense* (blueberry) [40], in accordance with our observation of *MYB4*′s downregulation in roots by salt (Figure 5).

Previous studies have shown that plants’ salt tolerance (acclimation, phenotypic plasticity or environmental difference) depends on developmental, physiological and metabolic processes [41,42,43,44]. Thus, we expected the MYB genes involved in wheat’s salt stress responses to have diverse functional roles [29]. 

MYB13 identified in our study regulates target genes with molecular functions linked to DNA binding transcription factor activity and metal ion binding that play key roles in diverse cellular processes, such as root development, suppression of gene expression and specification of plant organ identity (Table 2) (Chen et al., 2020). MYB3 has been previously described as the anthocyanin regulatory C1 protein, which responds to salt stress and presumably participates in a reported increase of approximately 35% in anthocyanin levels following exposure to 100 mM NaCl in purple wheat [45]. In our study, we observed upregulation of *MYB3* in both shoot and roots of BARI Gom-25 plants under prolonged exposure to this stress (Figure 5). In addition, correlations have been found in the presence of 100 and 200 mM NaCl but not in the absence of salt stress between the expression of *chalcone-flavanone isomerase (Chi-1)*, which is involved in the early stages of anthocyanin biosynthesis, and *flavanone 3-hydroxylase (F3h-1)*, the major regulatory checkpoint in anthocyanin synthesis [46]. Thus, earlier studies have shown that anthocyanin-related genes are co-expressed under salt stress and could participate in the initiation of salt stress tolerance signalling via MYB3. 

Genes regulated by MYB3 linked to salt stress encode the *zinc finger protein ZAT11* and *transcription factor HHO5* (Table 2). We observed upregulated expression of *zinc finger protein ZAT11* in both roots and shoots under salt stress (Figure 6A). GO data indicate that it participates in the regulation of several major physiological processes, including DNA-binding transcription factor activities, DNA-templated transcription and root development. It is also known that zinc finger proteins participate in abiotic salt tolerance, e.g., genes encoding the zinc finger proteins OsC3H33, OsC3H37 and OsC3H50 are reportedly induced by salt stress in rice [47]. Previous *in silico* analysis has also revealed increased expression of the *ZAT11* gene in salt-stressed root tissues [48]. Moreover, enhancement of salt tolerance mediated by changes in Na^+^ homeostasis and K^+^ acquisition has been observed in transgenic tobacco plants overexpressing a homolog, GhZFP1 [49]. Thus, the observed increase in the expression of *zinc finger protein ZAT11* in roots during salt stress could reflect a developmental response that is part of a stress avoidance strategy involving searches for less saline zones, while its observed expression in shoots could participate in ion homeostasis. 

Previous GO findings have revealed the roles of *transcription factor HHO5* linked, for example, to suppression of gene expression, transcription regulation and DNA-binding transcription factor activity (Table 2). Expression of *HHO5* reportedly increases in the first 6–12 h of salt stress in Arabidopsis, and it is most highly expressed in vegetative tissues, predominantly roots, according to an analysis using AtGenExpress [50]. Our data suggest that it may be more active at the onset of salt stress than during prolonged stress, as we detected no clear increase in its expression in shoots but downregulation in roots after 6 days of exposure to 100 mM NaCl (Figure 6B), correlating well with the observed expression pattern of *MYB4* (an *HHO5* regulator) in roots. Thus, *MYB4* could have a stronger regulatory influence than *MYB3* on *HHO5* at our sampling stage. 

We observed upregulation of *MYB4* in shoots and downregulation in roots under salt stress (Figure 5). *MYB4* has a known role in salt stress responses of *Nicotiana tabacum* (tobacco), as overexpression of *NtMYB4* suppresses the flavonoid biosynthetic pathway, which is upregulated under salt stress in wild-type plants [51]. Moreover, although MYB4 family members are expressed in germinating seeds and apical meristems of shoots and roots, tissue-specific expression analysis has shown that is expressed in most tissues and most strongly in roots [48,52,53]. Previous studies have also shown that modification of cell wall composition can enhance plants’ salt tolerance [54,55], and one gene regulated by MYB4 is involved in cell wall modification (Table 2). Furthermore, the MYB4 family is involved in the regulation of genes involved in calcium ion binding, osmo-sensory and abscisic acid-activated signalling pathways, regulation of stomatal movement and inhibition of programmed cell death. Accordingly, osmotic adjustment through influx and efflux of ions via transmembrane transport proteins, supported by H^+^ pumps, is involved in salt tolerance [43], and vacuolar proton ATPase A1 is responsive to salt stress in sugar beet [56]. 

In addition to the *HHO5 transcription factor*, we found two other proteins that are regulated by MYB4 (Figure 6C-D) (Table 2). One, a *Casparian strip membrane protein*, is embedded in the plasma membrane and linked to cell wall modification. Casparian proteins are responsive to abiotic stress, and it has long been known that salt stress can facilitate and accelerate Casparian strips’ formation [57]. We observed strong upregulation of the *Casparian strip membrane protein* (Figure 6C) in accordance with Casparian strips’ putative role in preventing non-selective entry of ions into the stele via apoplastic pathways [58]. Thus, its upregulation could reinforce protection from the negative effects of apoplastic transport of Na^+^ and increases in root-to-shoot delivery of Na^+^. Interestingly, silencing of *ZmSTL1*, which encodes a dirigent protein (ZmESBL) localized to the Casparian strip domain in maize, increases the apoplastic transport of Na^+^ across the endodermis under salt stress, thereby raising root-to-shoot delivery of Na^+^ via the transpiration flow and transpiration-dependent salt hypersensitivity [59]. *MYB4* is not upregulated in roots (Figure 5), but it may still be an early signal during the onset of persistent salt stress or another mediator of salt stress signals that triggers the upregulation of the *Casparian strip membrane protein*. 

The final gene found to be regulated by MYB4 is *respiratory burst oxidase homolog protein F*, which has several potential roles, including (among others) involvement in the osmo-sensory and abscisic acid-activated signalling pathways, regulation of stomatal movement and calcium ion binding (Table 2). The protein is reportedly expressed more strongly in root tissues than in shoot tissues [48]. We also observed higher expression under salt stress in root tissue (Figure 6D). This may be due to the link with both osmo-sensory and abscisic acid-activated signalling pathways, which have known roles in roots’ salt stress tolerance. Priming wheat seeds with ABA has been shown to enhance salt tolerance [60]. Moreover, ABA accumulation strengthens cytosolic K^+^ and Na^+^ homeostasis, thereby enhancing the water status of *Zea mays* (maize) plants in response to salt stress [61]. The observed decrease in shoot tissues cannot be linked to processes such as stomatal closure that may help restore salt tolerance in shoot tissues by reducing transpiration. Instead, we suggest that if stomatal closure occurs, it is mediated by signalling involving respiratory burst oxidase homolog protein F in the roots, in line with previous findings of links between stomatal closure and respiratory burst oxidase homolog protein F homologs in *N. benthamiana* (benthi) and *Cucumis sativus L*. (cucumber) [62,63]. 

MYB13 is involved in DNA binding, transcription activity, sequence-specific DNA binding and regulation of DNA-templated transcription [64,65,66]. Tissue-specific analysis has shown that it is expressed more strongly in roots than in shoots and is upregulated in abiotic stress conditions. Previous studies with rice, maize, Tartary buckwheat and Arabidopsis have revealed links between salt tolerance and MYB13 [67,68,69]. Collectively, these results support our finding that *MYB13* is upregulated under salt stress (Figure 5).

We also found that MYB13 upregulates *Arginine decarboxylase 1*, which has carboxylase activity and is involved in arginine catabolic processes, as well as spermidine and putrescine biosynthetic pathways (Table 2). It is expressed mainly in root tissue but most strongly in spikes and during developmental stages. Reductions in polyamine formation have also been found in the Arabidopsis mutant spe2-1 under salt stress due to lower arginine decarboxylase activity, which reduces salt tolerance [70]. We found that *Arginine decarboxylase 1* was clearly upregulated in both root and shoot tissues (Figure 6E) in accordance with its reported functional areas in Arabidopsis. 

Another gene regulated by MYB13 is *calcium-dependent protein kinase 13*, which is involved in diverse processes, e.g., responses to water deprivation, intracellular signal transduction, protein autophosphorylation and positive regulation of salt stress responses (Table 2). Similarly, *calcium-dependent protein kinase 7* (*OsCDPK7*) is reportedly upregulated by salt stress in rice, where it participates in the protection of the root meristem and vascular tissues, as well as the maintenance of osmotic homeostasis in these tissues [71]. We detected no upregulation of *calcium-dependent protein kinase 13* in roots but a clear increase in shoots (Figure 6F), indicating that it could be linked to a faster response in roots at the onset of salt stress than in shoots. 

The MYB59 transcription factor participates in the regulation of DNA-templated transcription and sequence-specific DNA binding, and in silico analysis showed that it is potentially upregulated during salt stress, which was confirmed by our qPCR assay (Figure 5). Tissue-specific expression analysis based on RNA sequencing data has shown that its gene is more strongly expressed in root tissues and reproductive tissues under salt stress [48]. We found that MYB59 regulates four genes. *Zinc finger protein ZAT11* was regulated not only by MYB59 but also by MYB3 and clearly upregulated in both root and shoot tissues, as discussed above (Figure 6A). *In silico* analysis of MYB59 identified another target gene, encoding *2-oxoglutarate-dependent dioxygenase*, which has the same expression patterns as *ZAT 11* (Figure 6G), is salt-responsive, has metal ion binding and dioxygenase activities and participates in flavonoid biosynthesis [51,72]. Additionally, *2-oxoglutarate-dependent dioxygenase* is also involved in rhizobial interactions and salt tolerance of *Glycine max* (soybean). Moreover, 32 phosphoproteins reportedly participate in the regulation of flavonoid synthesis or trafficking, including members of nine MYB families, e.g., GmMYB183, which is also involved in soybean salt tolerance [51,72,73]. 

Two other MYB59 target genes, encoding the *homeobox-leucine zipper protein HOX19* and *9-cis-epoxycarotenoid dioxygenase NCED5*, showed opposite expression patterns to the other target genes of MYB59 with downregulation in both root and shoot tissues under salt stress (Figure 6H-I). HOX19 is linked to transcription regulation and DNA-templated reactions (Table 2). In *Lophopyrum elongatum* (wheatgrass), there are strong indications that HOX19 is involved in its salt tolerance linked to roots [74]. However, we did not observe upregulation of the corresponding gene in either roots or shoots (Figure 6H), so its function in wheat requires further study. 

NCED5 is linked to the ABA biosynthesis pathway and carotene catabolism. Foliar application of β-carotene can help protect *Lepidium sativum* L (garden cress) plants from salt stress by contributing to processes including ion uptake regulation, reduction of H_2_O_2_ and malondialdehyde levels and increases in enzymatic antioxidant activities [75]. In rice plants, *NCED5* is clearly induced by salt stress, as [76] found that a *nced5* mutant had reduced ABA contents and impaired tolerance of both salt and water stress, while *NCED5* overexpression increased their ABA contents and salt tolerance. In addition, salt stress (150 mM NaCl) reportedly upregulates *NCED35* in Arabidopsis but impairs seed germination due to enhanced ABA biosynthesis and signalling [77]. Moreover, *in silico* analysis indicates that NCED5 is involved in hyperosmotic salinity responses [78]. This could explain our observation of low *NCED5* expression after 6 days of incubation with 100 mM NaCl (Figure 6I). Thus, the decreased expression we observed might be due to *NCED5* acting in the early stages of salt exposure rather than persistently in a stable saline environment where ABA and β-carotene levels may be controlled by other regulators. 

It should be noted that the downregulated proteins we observed could potentially have a diurnal expression pattern, e.g., with low expression at our sampling time point (2–3 h after onset of light) and higher expression in later parts of the day. They could also potentially be expressed strongly but transiently at the initiation of salt stress. Thus, further assays are needed to exclude (or confirm) these possibilities. 

## 5. Conclusions

In summary, we here put forward evidence that the transcription factors *MYB3*, *MYB4*, *MYB13* and *MYB59* are involved in wheat’s salt stress response, as they all are upregulated in both roots and shoots (except *MYB13* in roots) and act on several downstream target genes linked to salt stress. For example, the MYB59 target *2-oxoglutarate-dependent dioxygenase* was consistently upregulated at all sampling points under salt stress and participated in processes that are strongly associated with salt tolerance. The acquired knowledge of MYB transcription factors, their domains and binding specificities provides some insights into the regulation of plant stress responses generally and the regulation of wheat’s salt stress responses specifically. Understanding MYB transcription factors’ roles in salt tolerance is important for the rational development of salt-tolerant wheat, which is crucial to meet the needs to feed a growing population under climate change. Inter alia, further work is required to fully elucidate the roles of the identified MYB transcription factors and the functional genes they regulate.

## Figures and Tables

**Table 1:** Structural homologue hits for the MYB3, MYB4, MYB13 and MYB59 proteins.

Protein	Hits-PDBID	Identity (%)	RMSD (Å)
MYB3	6KKS:A	61	0.8
MYB4	6KKS:A	65	0.9
MYB13	6KKS:A	56	1
MYB59	6KKS:A	56	1.05

**Table 2:** Target genes involved in salt stress regulated by MYB proteins. Proposed functionalities, processes and cellular components based on Gene Ontology as given from http://wheat.cau.edu.cn/TGT/m21/?navbar=ByGeneID [31].

Gene, MYB Regulator	Function	Molecular Function	Biological Process	Cellular Component
*TraesCS3D02G350100* MYB3, MYB59	Zinc finger protein ZAT11 (uncharacterized)	DNA-binding transcription factor activity, metal ion binding	Regulation of transcription, DNA-templated, response to chitin, cellular response to nickel ion, regulation of root development	Nucleus
*TraesCS5D02G411800* MYB3, MYB4	Transcription factor HHO5	DNA-binding, DNA-binding transcription factor activity	Regulation of transcription, DNA-templated, negative regulation of gene expression, floral organ formation, specification of plant organ identity	Nucleus, cytosol
*TraesCS2D02G379300* MYB4	Casparian strip membrane protein 1 (uncharacterized)	4 iron, 4 sulfur cluster binding	Cell–cell junction assembly	Plasma membrane, integral component of membrane, Casparian strip
*TraesCS3B02G314000* MYB4	Respiratory burst oxidase homolog protein F (uncharacterized)	Peroxidase activity, calcium ion binding, NAD(P)H oxidase activity	Respiratory burst involved in defence response, osmosensory signalling pathway, response to ethylene, abscisic acid-activated signalling pathway, ethylene-activated signalling pathway, regulation of stomatal movement, carbohydrate homeostasis, negative regulation of programmed cell death, hydrogen peroxide biosynthetic process, defence response by callose deposition, oxidation-reduction process	Plasma membrane, integral component of membrane
*TraesCS7D02G063900* MYB13	Arginine decarboxylase 1	Arginine decarboxylase activity, cell wall modification	Arginine catabolic process, spermidine biosynthetic process, response to cold, putrescine biosynthetic process from arginine	NA
*TraesCS2A02G456100* MYB13	Calcium-dependent protein kinase 13 (uncharacterized)	Calcium ion binding, calmodulin binding, ATP binding, calcium-dependent protein serine/threonine kinase activity	Response to cold, response to water deprivation, peptidyl-serine phosphorylation, intracellular signal transduction, protein autophosphorylation, positive regulation of response to salt stress	Nucleus
*TraesCS4B02G19500* MYB59	Probable 2-oxoglutarate-dependent dioxygenase At3g111800	Metal ion binding, dioxygenase activity	Flavonoid biosynthetic process	NA
*TraesCS4D02G236600* MYB59	Homeobox-leucine zipper protein HOX19	Sequence-specific DNA binding	Regulation of transcription, DNA-templated	Nucleus
*TraesCS5B02G029300* MYB59	9-cis-Epoxycarotenoid dioxygenase NCED5, chloroplastic (uncharacterized)	Carotenoid dioxygenase activity, 9-cis-epoxycarotenoid dioxygenase activity, metal ion binding	Abscisic acid biosynthetic process, carotene catabolic process	Chloroplast, chloroplast stroma

## Data Availability

The data that support the findings of this study are available from the corresponding author upon reasonable request.

## Supplementary Materials

The following supporting information can be downloaded at: https://www.mdpi.com/article/10.3390/cells12101431/s1, Table S1: Primers used in qPCR expression analyses. Table S2: MYB multi-domain architectures divided into 28 groups with indicated numbers (No.) of members (585 in total). Table S3: PFAM domains identified in MYB transcription factor proteins. Table S4: The alignment of 719 MYB protein sequences used for constructing the phylogenetic tree. Table S5: Number of MYB domains identified for each of the genes used for the tree. Table S6: The phylogenetic tree file.
