# Supplementary material for: Genome-Wide Analysis of MYB Transcription Factors in the Wheat Genome and Their Roles in Salt Stress Response
Source: Cells. 2023 May 20;12(10):1431. doi: 10.3390/cells12101431 (PMC10217287; doi:10.3390/cells12101431)
Supplement: Supplementary file 1 [file cells-12-01431-s001.zip › ZIP MYB/2302 Supplementary Table S1_S2_S3 MYB paper.pdf]

Supplementary Table S1. Primers used in qPCR expression analyses

Gene		Primer 5' to 3'
MYB3	TraesCS4B02G323300	GAACTACTGGAACAGCACCCTT
		CCCGCAGTTAGAGGAAAGC
MYB4	TraesCS5D02G335700	TCCCTCCTTTCTCCTGTGACTC
		CATCCTCACCCTTCTTCTTGTCT
MYB13	TraesCS3A02G535100	ATTCCATTCCGTTCCATTTCC
		CACAGCAGCTAGGCACCAA
MYB59	TraesCS3B02G612200	TCTTCCGTGTTCACATCTCCA
		GCTTCATGTCCTCCTGCTCC
Actin	TraesCS1A02G274400	TCTATTTTGGCCTCTCTTAGCAC
		TTTCCTGTACCCCTTATTCCTC
Zinc finger protein ZAT11	TraesCS3D02G350100	GCGGGATCTGTCTGGACCTG
		CAGTCTAACATGGCGAGCGC
Transcription factor HHO5	TraesCS5D02G411800	GCCACCTCCAGAAATACCGT
		GAGATCGCCCACCAGCATAA
Casparian strip membrane protein 1	TraesCS2D02G379300	TTCCCGGCCTTCCTGTTCCT
		TTACCGTGTCGCAGACGAGC
Respiratory burst oxidase homolog protein F	TraesCS3B02G314000	TGCCATTGTGTACGTGGCTC
		AGAGCCGGACAGCATAACTG
Arginine decarboxylase 1	TraesCS7D02G063900	TGCCGTACCTGTCGTTCG
		CTGCTCATACCGCTCGACT
Calcium-dependent protein 13	TraesCS2A02G456100	ATCTTATGGATGCGGCGGAC
		GAAAAGGCTGCCACCAGATG
(Probable) 2-oxoglutarate-dependent dioxygenase (At3g111800)	TraesCS4B02G195000	TCAAGAGCCACGACAAGTGG
		TTTGATACCCTCCGGCACAG
Homeobox-leucine zipper protein HOX19	TraesCS4D02G236600	CCCTCAACCCGAAGCAGAAA
		TTCAGGAACTCGCAGTCCAC
9-cis-epoxycarotenoid dioxygenase NCED5, (chloroplastic)	TraesCS5B02G029300	ATTGCCTCGCCGCAGAAG
		AGGACATTGTGGGTGAACCCC

Supplementary Table S2. MYB multi domain architectures divided into 28 groups with indicated numbers (No.) of members (585 in total)

Group (No.)	PFAM accession, domain									
1 (363)	PF00249 Myb_DNA-binding	PF00249 Myb_DNA-binding	PF13921 Myb_DNA-bind_6	PF13921 Myb_DNA-bind_6						
2 (55)	PF00249 Myb_DNA-binding	PF13921 Myb_DNA-bind_6								
3 (44)	PF14379 Myb_CC_LHEQLE	PF00249 .Myb_DNA-binding								
4 (28)	PF00249 Myb_DNA-binding	PF00249 Myb_DNA-binding	PF13921 Myb_DNA-bind_6							
5 (15)	PF00249 Myb_DNA-binding	PF00072 Response_reg								
6 (12)	PF00249 Myb_DNA-binding	PF00249 Myb_DNA-binding	PF00249 Myb_DNA-binding	PF13921 Myb_DNA-bind_6	PF13921 Myb_DNA-bind_6	PF13921 Myb_DNA-bind_6				
7 (10)	PF00538 Linker_histone	PF00249 Myb_DNA-binding	PF13921 Myb_DNA-bind_6							
8 (9)	PF00249 Myb_DNA-binding	PF13921 Myb_DNA-bind_6	PF04433 SWIRM	PF16495 SWIRM-assoc_1						
9 (8)	PF00249 Myb_DNA-binding	PF15963 Myb_DNA-bind_7								
10 (7)	PF00249 Myb_DNA-binding	PF00249 Myb_DNA-binding	PF13921 Myb_DNA-bind_6	PF13921 Myb_DNA-bind_6	PF06640 P_C					
11 (6)	PF11831 Myb_Cef	PF00249 Myb_DNA-binding	PF00249 Myb_DNA-binding	PF13921 Myb_DNA-bind_6	PF13921 Myb_DNA-bind_6					
12 (3)	PF00249 Myb_DNA-binding	PF00249 Myb_DNA-binding	PF13921 Myb_DNA-bind_6	PF13921 Myb_DNA-bind_6	PF0664 P_C	PF06640 P_C				
13(3)	PF00249 Myb_DNA-binding	PF00249 .Myb_DNA-binding								
14 (3)	PF00249 Myb_DNA-binding	PF13921 Myb_DNA-bind_6	PF04433 SWIRM	PF16495 SWIRM-assoc_1	PF00569 ZZ					
15 (3)	PF00439 Bromodomain	PF00249 Myb_DNA-binding								
16 (2)	PF00249 Myb_DNA-binding	PF00249 Myb_DNA-binding	PF00249 Myb_DNA-binding	PF13921 Myb_DNA-bind_6	PF13921 Myb_DNA-bind_6					
17 (2)	PF00646 F-box	PF12937 F-box-like	PF00249 Myb_DNA-binding							
18 (2)	PF00249	PF00569								

	Myb_DNA-binding	ZZ								
19 (2)	PF00538 Linker_histone	PF00249 Myb_DNA-binding								
20 (1)	PF00646 F-box	PF12937 F-box-like	PF00249 Myb_DNA-binding	PF13921 Myb_DNA-bind_6						
21 (1)	PF00249 Myb_DNA-binding	PF13921 Myb_DNA-bind_6	PF04433 SWIRM							
22 (1)	PF00249 Myb_DNA-binding	PF13921 Myb_DNA-bind_6	PF13921 Myb_DNA-bind_6							
23 (1)	PF00249 Myb_DNA-binding	PF00249 Myb_DNA-binding	PF00249 Myb_DNA-binding	PF00249 Myb_DNA-binding	PF00249 Myb_DNA-binding	PF13921 Myb_DNA-bind_6	PF13921 Myb_DNA-bind_6	PF13921 Myb_DNA-bind_6	PF13921 Myb_DNA-bind_6	
24 (1)	PF00249 Myb_DNA-binding	PF00249 Myb_DNA-binding	PF13921 Myb_DNA-bind_6	PF13921 Myb_DNA-bind_6	PF18052 Rx_N					
25 (1)	PF00249 Myb_DNA-binding	PF00249 Myb_DNA-binding	PF13921 Myb_DNA-bind_6	PF13921 Myb_DNA-bind_6	PF0093 NB-ARC	PF18052 Rx_N				
26 (1)	PF00249 Myb_DNA-binding	PF00249 Myb_DNA-binding	PF00249 Myb_DNA-binding	PF00249 Myb_DNA-binding	PF00249 Myb_DNA-binding	PF13921 Myb_DNA-bind_6	PF13921 Myb_DNA-bind_6	PF13921 Myb_DNA-bind_6	PF13921 Myb_DNA-bind_6	PF13921 Myb_DNA-bind_6
27 (1)	PF00249 Myb_DNA-binding	PF00249 Myb_DNA-binding	PF00249 Myb_DNA-binding	PF00249 Myb_DNA-binding	PF13921 Myb_DNA-bind_6	PF13921 Myb_DNA-bind_6	PF13921 Myb_DNA-bind_6	PF13921 Myb_DNA-bind_6		
28 (1)	PF00249 Myb_DNA-binding	PF04433 SWIRM								

Supplementary Table S3. **PFAM domains identified in MYB transcription factor proteins.**

PFAM accession/domain	Description, reference	No.
PF00439 Bromodomain	Present in proteins involved in a wide range of functions such as acetylating histones, remodeling chromatin, and recruiting other factors necessary for transcription, thus playing a critical role in the regulation of transcription, PUBMED:24704920	1
PF00646 F-box	Mediating protein-protein interactions in a variety of contexts, such as polyubiquitination, transcription elongation, centromere binding and translational repression, PUBMED:8706131	1
PF12937 F-box-like		1
PF00538 Linker_histone	Histone proteins have central roles in both chromatin organisation (as structural units of the nucleosome) and gene regulation (as dynamic components that have a direct impact on DNA transcription and replication).	2
PF14379 MYB_CC_LHEQLE	This family is found towards the C-terminus of MYB-CC type transcription factors, and carries a highly conserved LHEQLE sequence motif.	1
PF11831 MYB_Cef	This family is a region of the MYB-Related Cdc5p/Cef1 proteins, in fungi, and is part of the pre-mRNA splicing factor complex.	1
PF13921 MYB_DNA-bind_6	This family contains the DNA binding domains from MYB proteins, as well as the SANT domain family, PUBMED:8882580	37
PF15963 MYB_DNA-bind_7		3
PF00249 MYB_DNA-binding	This entry represents the MYB domain found in transcription factor TFIIIB component B". The MYB-type HTH domain is a DNA-binding, helix-turn-helix (HTH) domain of approximately 55 amino acids, typically occurring in a tandem repeat in eukaryotic transcription factors, PUBMED:3185713	57
PF00931 NB-ARC	This is the NB-ARC domain, a novel signalling motif found in bacteria and eukaryotes, shared by plant resistance gene products and regulators of cell death in animals, PUBMED:9545207	1
PF06640 P_C	This family represents the C-terminus of plant P proteins. The maize P gene is a transcriptional regulator of genes encoding enzymes for flavonoid biosynthesis in the pathway leading to the production of a red phlobaphene pigment [1] and P proteins are homologous to the DNA-binding domain of MYB-like transcription factors [2], PUBMED:8768374, PUBMED:8313474	3
PF00072 Response_reg	This domain receives the signal from the sensor partner in bacterial two-component systems. It is usually found N-terminal to a DNA binding effector domain.	1
PF18052 Rx_N	This entry represents the N-terminal domain found in many plant resistance proteins, PUBMED:24194517	2
PF04433 SWIRM	This SWIRM domain is a small alpha-helical domain of about 85 amino acid residues found in chromosomal proteins. It contains a helix-turn helix motif and binds to DNA, PUBMED:16461455	6
PF16495 SWIRM-assoc_1	Much of the higher eukaryote SWI/SNF complex subunit SMARCC2 proteins is of low-complexity and or disordered. However, there are several short regions that are quite highly conserved. This is one of these regions. The function of the individual regions is not known.	3
PF00569.19 ZZ	Zinc finger present in dystrophin, CBP/p300. ZZ in dystrophin binds calmodulin. Putative zinc finger; binding not yet shown. Four to six cysteine residues in its sequence are responsible for coordinating zinc ions, to reinforce the structure, PUBMED:8848831	4
